# Rationale and study protocol of the EASY Minds (Encouraging Activity to Stimulate Young Minds) program: cluster randomized controlled trial of a primary school-based physical activity integration program for mathematics

**DOI:** 10.1186/1471-2458-14-816

**Published:** 2014-08-08

**Authors:** Nicholas Riley, David R Lubans, Kathryn Holmes, Philip J Morgan

**Affiliations:** Priority Research Centre in Physical Activity and Nutrition, School of Education, University of Newcastle, Callaghan Campus, Callaghan, Australia; School of Education, Faculty of Education and Arts, University of Newcastle, Callaghan, NSW 2308 Australia

**Keywords:** Physical activity, Primary school, Mathematics, On task behaviour, Accelerometry, Randomised controlled trial

## Abstract

**Background:**

Novel strategies are required to increase school-based physical activity levels of children. Integrating physical activity in mathematics lessons may lead to improvements in students’ physical activity levels as well as enjoyment, engagement and learning. The primary aim of this study is to evaluate the impact of a curriculum-based physical activity integration program known as EASY Minds (Encouraging Activity to Stimulate Young Minds) on children’s daily school time physical activity levels. Secondary aims include exploring the impact of EASY Minds on their engagement and ‘on task’ behaviour in mathematics.

**Methods/Design:**

Grade 5/6 classes from eight public schools in New South Wales, Australia will be randomly allocated to intervention (n = 4) or control (n = 4) groups. Teachers from the intervention group will receive one day of professional development, a resource pack and asked to adapt their lessons to embed movement-based learning in their daily mathematics program in at least three lessons per week over a six week period. Intervention support will be provided via a weekly email and three lesson observations. The primary outcomes will be children’s physical activity levels (accelerometry) across both the school day and during mathematics lessons (moderate-to-vigorous physical activity and sedentary time). Children’s ‘on-task’ behaviour, enjoyment of mathematics and mathematics attainment will be assessed as secondary outcomes. A detailed process evaluation will be undertaken.

**Discussion:**

EASY Minds is an innovative intervention that has the potential to improve key physical and academic outcomes for primary school aged children and help guide policy and practice regarding the teaching of mathematics.

**Trial registration no:**

Australian and New Zealand Clinical Trials Register ACTRN12613000637741 13/05/2013.

## Background

Global estimates demonstrate that less than 20% of young people are achieving the guidelines of 60 minutes per day of ‘health enhancing’ moderate-to-vigorous physical activity (MVPA) [1]. This is of concern as multiple physical and psychological health benefits can be attained when children are physically active [2]. While schools have long been identified as important institutions for the promotion of physical activity (PA) among children [3, 4], children’s time at school is commonly characterised by low levels of PA. Moreover, children also experience prolonged bouts of sitting while at school [5]. Reducing sitting time or sedentary behaviour has important and independent health implications for children [6]. Studies have found that sedentary behaviour is associated with a higher risk of overweight [7], adverse metabolic markers [8] and poorer mental health [9]. Therefore, reducing sitting time and promoting physical activity across the school day may have important health benefits for children [10, 11].

The crowded school curriculum, competing demands on teachers, low levels of teacher expertise in PA promotion and restrictive school policies have impacted on both the quality and quantity of PA opportunities in primary schools [12, 13]. Indeed, educational researchers have stated that that the single biggest barrier to PA promotion are teachers’ own beliefs, perceptions and attitude towards PA [14]. Despite these challenges, schools provide an ideal setting in which changes can be implemented for facilitating PA opportunities and reducing sedentary behaviour [15]. However, novel strategies for PA promotion throughout the school day that are feasible and appealing for teachers and schools to implement are needed [12, 16, 17]. One such strategy is PA integration across the curriculum [4, 14, 18].

The potentially appealing aspect for teachers and schools of integrating PA across the school curriculum is that the benefits to children extend beyond the health benefits of physical activity [19, 20]. For example, recent research suggests that movement aids learning and that the integration of PA across the curriculum may enhance learning in other curriculum areas [20, 21]. This challenges the belief that schools need to increase academic time and reduce PA time to improve academic performance [22]. There is also an increasing body of literature that focusses on the association between PA and academic performance and provides evidence that PA enhances children’s cognitive functioning, concentration and on-task behaviour [23]. The integration of PA into other subjects may also enhance connectedness by providing real life application of academic concepts to enable students to view learning as significant and meaningful [24]. For example, in mathematics, using real stimuli such as stopwatches, tape measures, and trundle wheels to gather data provides a real life context and interactive teaching methods that promote movement, which are associated with greater learning [24].

The proposed program builds on a successful pilot of the EASY (Encouraging Activity to Stimulate Young) Minds program [25], where significant intervention effects were found for MVPA and sedentary time for the intervention group during mathematics lessons and across the whole school day. Furthermore, children displayed significantly greater ‘on-task’ behaviour across the intervention period. However, the pilot study research was carried out in a single school and all sessions were planned and delivered by a member of the research team.

## Methods/Design

### Study design

The EASY Minds program is a 6 week primary school-based intervention and will be evaluated using a cluster randomised controlled trial (RCT). Ethics approval has been sought and obtained from the University of Newcastle, NSW, Australia and the New South Wales Department for Education and Communities (SERAP: 2013011).The EASY Minds trial is registered with the Australian and New Zealand Clinical Trials Registry (ACTRN12613000637741).

Following the initial recruitment processes, all eligible participants will complete baseline assessments. The design, conduct and reporting of the EASY minds program will adhere to the Consolidation Standards of Reporting Trials (CONSORT) guidelines and the extension for a cluster randomised control trials (RCT) [26]. Principals, teachers and parents will need to provide written informed consent.

### Recruitment and study participants

Eight government public primary schools from the Hunter Region, NSW, Australia will be recruited to participate in the EASY minds RCT. Stage 3 classes (Grades 5 and 6) at the study schools will be invited to participate in the study. School principals will receive an initial letter followed by an email. Schools will be randomly selected from a list of primary schools within a 20 km radius from the University of Newcastle. Schools will then be matched on size and demographics using the participating schools index of community socio-educational advantage (ICSEA). The ICSEA value is determined based on family background information provided to schools directly by families and includes data relating to parental occupation, and the school education and non-school education levels they achieved.

**Figure 1:**
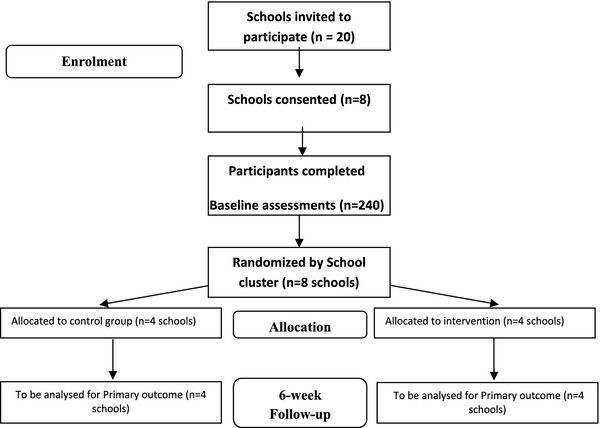
**Flow of participants through the EASY Minds study**

Randomization will occur after baseline assessments. A simple computer algorithm will be used to randomly allocate schools to either control or the treatment conditions by an independent researcher not involved in the study. This method will ensure all schools have an equal likelihood of allocation into one of the two study arms. Trained research assistants will conduct all assessments and administer all student questionnaires. All researchers will complete training sessions prior to assessment to maintain consistency and where possible, the same assessors will be used at baseline and post-test. Figure 1 shows the flow of participants through the study.

### Sample size calculation

Power calculations were conducted to determine the sample size required to detect changes in the in the primary outcome of accelerometer-determined physical activity counts per minute (CPM) [27]. Calculations assumed baseline-posttest correlation scores of *r* = 0.30 and were based on 80% power, with alpha levels set at *p* < 0.05. Using the standard deviation (SD) of change observed in the EASY Minds pilot study (SD =200 CPM) and a conservative intra-class correlation coefficient (ICC = 0.15), it was calculated that a study sample of N = 200, with 8 clusters (i.e., schools) of 25 students would provide adequate power to detect a between group difference of 200 CPM across the school day.

#### Intervention

The EASY Minds program will involve teachers adapting mathematics lessons over a 6 week (3 × 60 min sessions per week) period to ensure movement-based learning is applied to the NSW K-10 Mathematics syllabus [28]. The intervention program will involve a 1 day teacher professional learning day, the provision of equipment and resources, teacher-initiated adaptation of mathematics lessons to incorporate movement-based learning and support for teachers during program implementation.

All classroom teachers from the intervention schools will be invited to attend a 6 hour professional learning workshop conducted at the university and delivered by NR, KH and PJM. The content of the professional learning day is outlined in Table 1. The one day workshop includes a rationale for PA integration, presentation of results from the feasibility trial, practical examples of PA integration and a peer supported planning session.

**Table 1:** **EASY Minds Professional Development workshop**

Session	Focus	Content
**1**	Theory Introduction and EASY Minds program objectives	* Research on the effects of a school-based program on physical activity and academic performance.
		* Introduction to the EASY Minds program and key measures.
		* Mathematics: Student engagement in mathematics
		* Managing the learning environment
**2**	Practical Movement-based learning. Practical considerations and key teaching principles.	* Introduction to practical activities that promote mathematical concepts
		* Teachers will expand their repertoire of teaching practices by learning about and participating in activities successfully trialed in schools in advance.
		* Links to key elements of Quality Teaching Framework e.g. quality learning environment Engagement, high expectations, social support, self-regulation.
	Key teaching principles of movement-based learning	* Watching and discussion of previously recorded video footage of movement-based learning lessons filmed by and delivered by research team.
**3**	Theory Planning and delivery	* Teachers will plan an EASY Minds enhanced mathematics unit of work and individual activities using their current mathematics unit of work using both previously prepared resources, knowledge acquired and peer support.
		* Teachers will be instructed in how to utilise current resources and how to embed resource kit provided into their teaching.
**4**	Practical	* Teachers will deliver to their peers both an indoor and outdoor movement-based activity from their lesson plan developed in previous session.
		* Feedback and support from peers via observation and discussion.
**5**	Conclusion	* Recap of key principles of movement-based lessons.
		* Introduction to fidelity checking procedures.
		* Explanation of email support by research team.

The workshop is designed to engage, inspire and equip the teachers with the necessary skills to plan and deliver movement-based mathematics lesson. The professional learning day is registered and accredited with the New South Wales Institute of Teachers and attendees will be given professional learning hours towards their teacher accreditation. Attendees will be familiarized with the increasing evidence linking physical activity with academic performance and evidence related to the consequences of student disengagement from mathematics in the middle years of schooling. They will be provided with demonstrations and resources for learning experiences to promote physical activity across the primary school mathematics curriculum. These activities and resources were previously employed in the successful feasibility trial. The professional learning day will promote two types of mathematical lessons. i) Activities that use PA as a platform for the development of procedural fluency of fundamental number operations [28]. For example, students can recall multiplication tables whilst skipping, throwing and catching a ball or running through drill ladders. ii) Lessons that look at mathematics in the world around the school [29]. For example, estimating and measuring distance, finding shapes and identifying properties of them in the natural environment, data collection and representation involving fundamental movement skills of kicking throwing, striking. Example ideas can be found in Table 2.

**Table 2:** **E.A.S.Y. Minds: self evaluation checklist**

(***Please circle and provide comments)***		***(1 = Not at all true***to ***5 = Very true)***				
Mathematical concepts	i) Key mathematical concepts were reinforced throughout the movement-based activity	1	2	3	4	5
	ii) Movement aided and promoted learning	1	2	3	4	5
	iii) Students were given feedback to support their mathematical knowledge and understanding	1	2	3	4	5
Activity levels	i) Transitions were managed smoothly	1	2	3	4	5
	ii) Students assisted in the set-up and collection of equipment	1	2	3	4	5
	iii) Equipment used promoted physical activity	1	2	3	4	5
Engagement	i) Students were engaged by the activities taught	1	2	3	4	5
	ii) Students remained on-task throughout the lesson	1	2	3	4	5
	iii) Students enjoyed the movement-based mathematics lesson	1	2	3	4	5

Following the completion of the professional learning day, all schools will receive an EASY Minds equipment pack containing a selection of sporting and mathematical resources identified in the feasibility trial as being relevant for promoting movement-based learning. This includes, but is not limited to, stopwatches, tape measures, large dice, drill ladders, basketballs, skipping ropes, numbered beanbags, target mats and numbered flexi-domes (value $AU800). All participating teachers will receive a CD-ROM with example movement-based activity descriptions developed by the research team at the culmination of the professional learning day to be used as a guide when integrating PA across their existing mathematics program. These activities are aligned with the current NSW mathematics syllabus An important component of the program is a focus on teacher ownership and a tailored approach to meeting individual school requirements regarding each schools own specific unit of work and as such, the content of lesson material needs to be individually planned and prepared. The professional learning sessions will be delivered by academics (NR, KH and PJM) who are experienced researchers in the fields of physical activity promotion, mathematics education and primary school pedagogy and qualified teachers. Teachers will be encouraged to be creative and to develop their own lessons, thereby developing ownership of the program and increasing the likelihood of sustaining the program beyond the intervention period [18].

A key principal of the EASY Minds program is the alignment of the program with the NSW Quality Teaching Framework [24]. The NSW quality teaching framework encourages teachers to develop innovative skills that promote high levels of intellectual quality, establish a quality learning environment and generates significance by making learning meaningful and purposeful [24].

The intervention will run for 6 weeks during the schools’ regular timetabled mathematics sessions and will be delivered by the regular classroom teacher, who will translate their knowledge from the professional development training to create movement based learning activities for mathematics.. Teachers will be encouraged to integrate PA in Mathematics sessions (60 minutes) on at least three occasions per week whilst maintaining the key focus on the desired mathematical outcomes from the current syllabus. The teachers will all receive a weekly email offering tips and strategies from the research team and a fortnightly fidelity check during weeks 1, 3 and 5 of the intervention.

### Outcomes

Evaluation of the EASY Minds program will involve a variety of instruments and surveys to report on physical activity and key academic variables (on task behaviour, mathematical performance and attitude towards mathematics). All assessments will be conducted by trained research assistants and carried out in a sensitive manner. The collection of height and weight data will be measured behind a portable screen by gender matched researchers. All PA and academic measures will be measured at baseline and post-test (6 weeks).

### Physical activity

The primary outcome will be children’s school-based PA levels. Actigraph accelerometers (GT3X, Pensacola, USA) will be used to provide an objective measure of both PA intensity and duration [30]. The Actigraph accelerometer has acceptable reliability and validity in both children and adolescents [31]. Accelerometers will be worn Monday through to Friday, during school hours only. This will vary slightly for each school setting as the schools are likely to have different start and finish times. The classroom teachers will receive training in how to instruct students to wear accelerometers and be responsible for distributing and collecting the accelerometers on a daily basis. Accelerometers will be attached to an adjustable elastic belt and worn on the right hip. Raw data from the accelerometer will be screened and analysed using Meter plus software version 4.7 which allows for time specific analysis to accurately analyse lesson- and school-time PA. Participants’ PA will be included for analysis if they wear the accelerometer for at least five school hours on any given day. Similarly, students will only be included in the analysis if they wear the accelerometer for 50 minutes of the 60 minute mathematic lessons. This time period may vary from school to school as a result of schools individual timetables. The Evenson cut-points will be used to classify activity as sedentary: (0–100 CPM, light (101–2295 CPM), moderate (2296–4011 CPM), vigorous 4012 - ∞ CPM ) or MVPA [32]. MVPA is a variable calculated by summing moderate and vigorous PA. Data will be collected in 15 second epochs and non-wear time will be defined as 20 minutes of consecutive zero’s [33].

### Academic measures

#### On-task behaviour

Children’s on-task behaviour will be observed using a momentary time sampling procedure. This observational tool has been adapted from the Behaviour Observation of Students in Schools [34] and the Applied Behaviour Analysis for Teachers [35]. Six students per class group of either sex will be selected and observed in 15 second intervals on a rotational basis over a 30 minute period in the allocated mathematics time slot. A cross-section of students with varying mathematical ability will be selected by the classroom teacher from those working above, those working at and those working below the class average as determined by exiting teacher assessments. On-task behaviour will include behaviour that could be categorised as being ‘actively engaged’ or ‘passively engaged’. Actively engaged refers to a child being actively engaged in academic responding, e.g. reading, writing, performing a set task. Passively engaged will be categorised as behaviour where the child is listening to the teacher or a fellow student but is not actively participating in a set task. Off-task behaviour includes behaviour that can be described as being either: ‘off-task motor’ where a child moves in a manner not associated with the task, for example walking around the class; ‘off-task verbal’ includes non-work related talking or ‘off-task passive’ where a child is disengaged but passive, including staring into space. Two trained research assistant observers will observe simultaneously. This method of systematic observation has been recommended when seeking to simply describe the classroom behaviour of children [36]. Classroom behaviour will be reported as a percentage of time.

#### Attitude to mathematics

Participants’ attitudes to mathematics will be measured using a 24-item questionnaire containing two separate subscales: i) Confidence e.g. *I get good grades in maths*, and ii) Usefulness e.g. *Maths is a worthwhile necessary subject* [37]. Each scale consists of 12 items with six items positively worded and six negative. Studies on the psychometric properties of the scale provide evidence for the reliability and validity of the subscales [38].

#### Mathematic achievement

Mathematics achievement will be measured using a Mathematics Progressive Achievement Test (PAT) [39]. For the purpose of this study cohort, PAT version 3 will be used. The PAT Mathematics test has 37 questions and takes 40 minutes to administer. The test will be administered by the classroom teacher under exam conditions as recommended by the Australian Curriculum for Educational Research (ACER). The 37 questions form separate items for individual mathematics sub-strands. These being number (n = 14), Space (n==6), Measurement (n = 6), Data (n = 5) and Number (no calculator) (n = 6). The test will provide evidence of student’s strengths and weaknesses and change over time.

Demographic information (i.e., age, sex, language, country of birth) will be collected at baseline via questionnaire alongside questionnaires of children’s preferred learning styles and intelligence strengths. These will be used to profile the children recruited in the study. Learning styles will be measured using the Barsch Learning Style Reference Form [40] and students’ preferred perceptual modality will be determined. Preferred perceptual modality (learning style) has been defined as the conditions under which individuals concentrate process and internalise information [41]. The Barsch learning style reference form defines three perceptual modalities. These are kinaesthetic (relating to body movement), visual (relating to the eyes) and auditory (relating to the ears). Students’ intelligence strengths will be assessed using a Multiple Intelligences Checklist for Upper Primary and Secondary (MICUPS) [42].

Weight will be measured in light clothing without shoes using a portable digital scale (Seca 770, Wedderburn) to the nearest 0.1 kg and height will be measured to the nearest 0.1 cm using a portable stadiometer (Design No. 1013522, Surgical and Medical Products, Seven Hills, Australia). Height, weight, learning style and intelligence strengths will only be measured at baseline to profile the sample. No measures will be taken at follow up.

### Process evaluation

The overall feasibility of the EASY Minds program will be examined using a number of metrics to form a detailed process evaluation. Measures of recruitment, retention, adherence and satisfaction from teachers and students will be collected. We will also use a semi-structured discussion framework to conduct focus groups with students and one-on-one interviews with teachers. All questionnaires and focus group interviews for both students and teachers will be conducted by members of the research team. Teachers attending the professional learning session will complete a short evaluation questionnaire, which will assess teachers’ perceptions of the skills and ideas gained from the training, their satisfaction with the quality of the teacher training and their confidence to plan and deliver movement-based mathematics lessons across the study period. A 5-point Likert scale will be used with responses ranging from ‘*strongly disagree’* = 1 to ‘*strongly agree’ = 5*.

For example, i) the practical session improved my confidence to teach PA mathematics lessons, ii) the workshop provided me with useful information and skills that may improve my teaching. Additionally, participants will be asked for suggestions to improve the learning workshop or the program principles to assist further in the teaching of movement-based learning.

Throughout the intervention period, teachers will be asked to complete an activity evaluation log after each session and reflect on their lesson and rate their lesson using a 5-point Likert scale. The teachers will all receive a weekly email offering tips and strategies from the research team and a fortnightly fidelity check via observation during weeks 1, 3 and 5 of the intervention. During this observation, a five minute discussion will take place where teacher and researcher will discuss a self-evaluation/ activity log focused on 3 items. These will be 1) mathematical concepts (n = 3), e.g.; the key mathematical concepts reinforced throughout the movement based activity, 2) activity levels (n = 3) e.g. transitions were manage smoothly and 3) engagement (n = 3) e.g. students were engaged by the activities taught. (See Table 3).

**Table 3:** **Example activities from professional learning day**

Mathematics content	Movement-based lesson
Using an Empty number line	• Students are encouraged to use a number line drawn in chalk outside and utilise the jump strategy.
	• Present the students with a number problem. E.g. 8000–673.
	• Students should try to complete the number line in the most efficient way.
	• Assign each “jump” a physical activity. Students can create their own movement
	• 1000 = Squat, 100 = jump, 10’s = lunge, 1’s = bottom kicks.
	• In this case the answer would be 7327. Students would perform 7 squats, 3 jumps, 2 lunges and 7 bottom kicks.
	Students can be presented with a series of operations and be encouraged to use an empty number line.
Multiplication and Division	• Students will throw up to 5 bean bags on to a numbered target. They add up the total. They then divide the total by the number thrown. This will give the mean score.
	• Each child throws two bean bags on to the target. They then roll the 20 sided dice and multiply the number rolled by the total score.
	• Children should be encouraged to estimate their answer and record before actually working out.
Recognising Factors, multiples and prime numbers	• Arrange numbered flexi domes throughout the area with the numbers in random order
	• Students run/skip/hop/side gallop etc. to the flexi dome applicable when the scenario is given.
	• What is one factor of 40? Repeat this question but change the number e.g. 75, 16, 84 etc.
	• Show me a factor of 24, and then hop to the pair of the
	• Find multiples of the number 3.
	• Find a prime number.
Three Dimensional Space	• Identify and describe the properties of three dimensional objects, for example number of faces, apex of a pyramid, number of edges etc.
	• Teachers can ask “How many vertices does a cube have?”
	• Students are to answer by skipping the required amount to answer the question. Students can ask each other and work in pairs.
Two Dimensional Space	Netball court or other marked pitch.
	• Working in small groups students are to classify all shapes they can identify on a netball court.
	• Students are to then draw and measure all key parts.
	• Students need to include length, width, radius, diameter, circumference, semi-circle and diagonals.
	• Using appropriate scale students are to draw an accurate scaled diagram

Upon completion of the 6-week program, all students will complete a process evaluation questionnaire. This questionnaire will be administered to determine students’ perceptions of integrating physical activity within the curriculum focusing on values of enjoyment and mathematical outcomes. A 5-point Likert scale will be used with responses ranging from ‘*strongly disagree’* = 1 to ‘*strongly agree’ = 5*. For example, “*I liked being physically active in Math’s****outside****the classroom*”.

#### Student focus groups and teacher interviews

Focus group interviews will be conducted with the students and phone interviews will be carried out with participating teachers. For the focus groups, two groups of six students per class (mixed sex) will be selected based on a range of mathematical abilities determined by the class teachers. Each teacher will be asked to select two children of higher, middle and lower ability for the class cohort. The 10–15 minute focus groups will use semi-structured questions. The focus group will be conducted by a researcher not directly involved in the current project.

The focus groups will be recorded and later transcribed by an independent third party. Specifically, the questions asked in the students’ groups will be designed to explore their perceptions of the EASY Minds outdoor mathematics lessons and associated activities, their mathematics lessons prior to and subsequent to their involvement in the program, as well as the students’ appraisal of how the EASY Minds lessons had influenced their perception of mathematics, and learning related to mathematical concepts The focus group discussion framework was designed to elicit responses to the following questions: How would you describe your math’s classes before the EASY Minds program? Did you enjoy this? Did you enjoy the outdoor EASY Minds math’s lessons? Why? Can you give me an example? Did this make the math’s activities more interesting? What kinds of activities did you enjoy doing in the E.A.S.Y. Minds program? Can you tell me if being active in math’s classes helped you learn? Why/ why not? If so can you give me an example?

Additionally, a one on one phone interview will be conducted with the teachers involved in the intervention group, after program completion. The 15 minute interview will be recorded and transcribed by an independent researcher. The interviews with teachers will be designed to elicit their perceptions of EASY Minds lessons compared to regular mathematics lessons. Teachers will also be asked to identify major challenges to the implementation of EASY Minds lessons, as well as their appraisal of learning outcomes and students’ enjoyment of the lessons, with particular emphasis on the role of physical activity in student engagement.

The following types of questions will be asked: Did you enjoy teaching an active mathematics session as opposed to a classroom based lesson? What were the major challenges to you as a teacher of active mathematics sessions? Do you think your students enjoyed the lessons/why/ why not? How well do you they think the students understood the mathematics content in the physically active lessons? Can you give me a specific example? Do you think the PA aspect of the lesson contributed to greater engagement in the lesson compared to how that same mathematics content would usually be taught?

### Statistical methods

Statistical analysis of both the primary and secondary outcomes will be conducted with linear mixed models using SPSS statistics version 20 and alpha levels will be set at *p* > 0.05.

The models will be used to assess the impact of treatment (EASY Minds or control), time (baseline and post-test) and the group-by-time interaction, these three terms forming the base model. The models will be specified to adjust for the clustered nature of the data and will include all randomized participants in the analysis. Mixed models are robust to the biases of missing data and provide appropriate balance of Type 1 and Type 2 errors [43]. Mixed model analyses are consistent with the intention-to-treat principle, assuming the data are missing at random [44]. Sex and weight status (based on body mass index) will be included as covariates in the models. Further sub group analysis will be conducted based on sex, weight status, mathematical ability, and enjoyment of mathematics and on-task behaviour.

The focus groups and interviews will be digitally recorded with the participants’ consent and transcribed verbatim. A computer program (NVIVO 10) will be used to assist with the organisational aspects of data analysis. Analysis will be conducted by an independent qualitative researcher. Analysis will be performed using a standard general inductive approach to qualitative analysis. Initially, inductively derived codes or labels will, be formulated from the meaning units arising from the data. The developing coding scheme will be continually revised and further expanded after coding of additional transcripts. Following coding of all the transcripts, emerging themes will be identified and defined.

## Discussion

The primary aim of this study is to evaluate the impact of a curriculum-based physical activity integration program known as EASY Minds on children’s daily school time PA levels. The secondary aim is to examine the impact of the program on student engagement and on-task behaviour in mathematics lessons and also determine program feasibility as it will be delivered by trained classroom teachers. The study will use a novel strategy in that it teaches current classroom practitioner’s to design their own lessons and integrate PA across the mathematics curriculum during traditional academic instruction time.

Previous school-based PA intervention studies have highlighted the importance of teacher behaviour on intervention outcomes [45]. A critical aspect of this study is that classroom teachers will be taught to deliver the intervention in the professional learning day. Comprehensive professional development has been identified in previous studies as a critical factor in improving the effects of school-based interventions [46]. Previous interventions have found teachers are willing to integrate PA into the academic subject, but lack the necessary skills and knowledge. Critically, this study is unique in that teachers will be given autonomy to plan and deliver their own lessons, using knowledge gained through the professional learning day. Teacher ownership of the program has the potential to lead to greater sustainability of the program and enable teachers to integrate PA across other curriculum areas.

A clear strength of this study is the rigorous process evaluation including quantitative and qualitative measures to explore program feasibility. Many other programs have reported issues with the intended delivery of the intervention as designed, thus effecting the true impact of the intervention [47]. Our detailed process evaluation will help us examine the views of participants (teachers and students), and help distinguish between an intervention that is poorly designed and one that may be poorly delivered [48]. This is necessary in this study due to the multisite delivery.

Enhancing student engagement may be particularly important for mathematics, as studies have demonstrated that student interest and attitudes towards an academic subject are a key predictor of academic success [49]. Attitude towards mathematics plays a significant role in mathematics achievement [50] and the development of negative attitudes, have long been a concern in mathematics education [51]. There is also growing evidence that subject boundaries within schools may act to inhibit innovation and the development of interdisciplinary skills such as problem solving, creativity, collaboration and self-regulation [52]. Ultimately this can lead to student disengagement, particularly evident in traditional academic subject areas like mathematics [53]. It is widely accepted that, by the end of Grade 6 (ages 12–13), students are developing lifelong attitudes towards mathematics [54] and that disengagement in mathematics is considered a factor in the declining trend in mathematical performance among students internationally [55]. Student enjoyment of mathematics is also recognised as a key ingredient for addressing student disengagement [56] and that attitudes towards mathematics are not stable and fixed [57], therefore innovative interventions, such as PA integration, may have the potential to positively affect attitudes and engagement [57].

A recent review of classroom-based PA interventions found that they are usually infrequent and often presented and analysed alongside whole school PA interventions (e.g., interventions targeting recess and lunch-time) [58]. This limitation has seen PA measures taken across the whole day and has not identified the specific impact during the actual intervention period. The need for well-designed interventions focussing on both health, PA and learning outcomes has been identified by the authors of the review, who also highlighted the need for teachers to act as agents of change and to be involved in the delivery of subsequent programs to improve the cost effectiveness, sustainability and feasibility of programs.

To our knowledge, no previous interventions have reported the effects of a classroom-based PA intervention on sedentary behaviour outcomes using accelerometery or examined sedentary time across the school day in mathematics. Significantly, our program will provide a unique approach as it uses PA within the curriculum to promote learning outcomes, provides objective measures of PA, while also measuring academic attitudes, on task behaviour and mathematical academic achievement. Furthermore, the program will be delivered by trained classroom teachers who will embed movement-based learning in their own classrooms in mathematics lessons. Other studies have provided actual materials to teachers to deliver [47]. A unique aspect of this study is that it will allow scope for teachers to plan and deliver their own lessons using the training day as a stimulus.

An additional study strength is the use of an objective measure of PA and will report on both MVPA and sedentary time and will provide evidence of the program on three key academic variables (attainment, attitude, on task behaviour). Accelerometers are advantageous when working with children because unlike self-report measures of PA, they help eliminate language and literacy difficulties, recall bias and social desirability bias [33]. Also as the monitors are to be worn across the school day, only compliance rates should be high, face-to-face distribution by the trained participating teacher will ensure the proper and consistent placing of the correct accelerometer on each participant [59], it is also likely that very few if any monitors will be lost. It has been reported that in previous study up to five percent of monitors may be lost if monitors are distributed by mail or worn across the whole day [59].

The findings of the EASY Minds RCT will provide valuable information for other research groups looking for evidence based research on PA across the school day and other key educational outcomes associated with mathematics in the primary school. Classroom based PA interventions are infrequent, and seldom published in peer reviewed journals. Importantly it is important for school-based PA interventions to report on both health and educational outcomes. EASY Minds has the potential to change school policy and practice in relation to PA integration, increase school time physical activity levels and enhance a range of key educational outcomes relating to mathematics.
